# A systematic comparison of genome-scale clustering algorithms

**DOI:** 10.1186/1471-2105-13-S10-S7

**Published:** 2012-06-25

**Authors:** Jeremy J Jay, John D Eblen, Yun Zhang, Mikael Benson, Andy D Perkins, Arnold M Saxton, Brynn H Voy, Elissa J Chesler, Michael A Langston

**Affiliations:** 1 The Jackson Laboratory, Bar Harbor, ME 04609, USA; 2 Oak Ridge National Laboratory, Oak Ridge, TN 37831, USA; 3 Pioneer Hi-Bred International Incorporated, Johnston, IA 50131, USA; 4 Linköping University, SE-581 85, Linköping, Sweden; 5 Mississippi State University, Mississippi State, MS 39762, USA; 6 University of Tennessee, Knoxville, TN 37996, USA

## Abstract

**Background:**

A wealth of clustering algorithms has been applied to gene co-expression experiments. These algorithms cover a broad range of approaches, from conventional techniques such as *k*-means and hierarchical clustering, to graphical approaches such as *k*-clique communities, weighted gene co-expression networks (WGCNA) and paraclique. Comparison of these methods to evaluate their relative effectiveness provides guidance to algorithm selection, development and implementation. Most prior work on comparative clustering evaluation has focused on parametric methods. Graph theoretical methods are recent additions to the tool set for the global analysis and decomposition of microarray co-expression matrices that have not generally been included in earlier methodological comparisons. In the present study, a variety of parametric and graph theoretical clustering algorithms are compared using well-characterized transcriptomic data at a genome scale from *Saccharomyces cerevisiae*.

**Methods:**

For each clustering method under study, a variety of parameters were tested. Jaccard similarity was used to measure each cluster's agreement with every GO and KEGG annotation set, and the highest Jaccard score was assigned to the cluster. Clusters were grouped into small, medium, and large bins, and the Jaccard score of the top five scoring clusters in each bin were averaged and reported as the best average top 5 (BAT5) score for the particular method.

**Results:**

Clusters produced by each method were evaluated based upon the positive match to known pathways. This produces a readily interpretable ranking of the relative effectiveness of clustering on the genes. Methods were also tested to determine whether they were able to identify clusters consistent with those identified by other clustering methods.

**Conclusions:**

Validation of clusters against known gene classifications demonstrate that for this data, graph-based techniques outperform conventional clustering approaches, suggesting that further development and application of combinatorial strategies is warranted.

## Background

Effective algorithms for mining genome-scale biological data are in high demand. In the analysis of transcriptomic data, many approaches for identifying clusters of genes with similar expression patterns have been used, with new techniques frequently being developed (for reviews see [1,2]). Many bench biologists become mired in the challenge of applying multiple methods and synthesizing or selecting among the results. Such a practice can lead to biased selection of "best" results based on preconceptions of valid findings from known information, which raises the question of why the experiment was performed. Given the great diversity of clustering techniques available, a systematic comparison of algorithms can help identify the relative merits of different techniques [3,4]. Previous reviews and comparisons of clustering methods have often concluded that the methods do differ, but offer no consensus as to which methods are best [3,5-9]. In this paper, we compare a broad spectrum of conventional, machine-learning, and graph-theoretic clustering algorithms applied to a high quality, widely used reference data set from yeast.

A popular and diverse set of clustering approaches that have readily available implementations were employed in this analysis (Table 1). These include five traditional approaches: k-means clustering [10], and the *de facto *standard hierarchical clustering, on which we tested four agglomeration strategies: average linkage, complete linkage, and the methods due to McQuitty [11] and Ward [12]. These approaches create clusters by grouping genes with high similarity measures together.

**Table 1:** Overview of algorithms tested

			Allows Overlapping Clusters			
				
				Pre-specified Number of Clusters (*k*)		
					
					Thresholded Correlations	
				
Method	Type	Result Range				Parameters Tested
Ward	Hierarchical	*k*		Y		Average cluster size
Average	Hierarchical	*k*		Y		Average cluster size
McQuitty	Hierarchical	*k*		Y		Average cluster size
Complete	Hierarchical	*k*		Y		Average cluster size
k-Means	Partitioning	*k*		Y		Number of clusters
SOM	Neural network	*k*		Y		Grid size/type^a^
QT Clust	Partitioning	24-385				Maximum cluster diameters
CAST	Graph-based	1-6162			Y	Threshold
CLICK	Graph-based	4-32				Cluster homogeneity
WGCNA	Graph-based	4-160				Power, Module detection method
NNN	Graph-based	23-52	Y^b^			Minimum neighborhood size
k-Cliques Communities	Graph-based	1-68	Y		Y	Threshold, Clique size
Maximal Clique	Graph-based	1,000-64,000	Y		Y	Threshold
Paraclique	Graph-based	8-615	Y^c^		Y	Threshold, Glom factor

Clustering methods are listed by name, along with the type of algorithm, and a general listing of parameters tested. Number of clusters in the result, given the parameters and data set tested, is only provided here as an approximate figure. Empty results are obviously not included. ^a^Grid type can be either rectangular or hexagonal, in an *m *x *n *layout. We tested both types, but used an *m *x *m *layout for simplicity (*k = m^2^*). ^b^Rarely occurs in practice. On this data set we observed no overlap with NNN. ^c^Optional, not used in this analysis.

Seven graph-based approaches are examined: k-clique communities [13], WGCNA [14], NNN [15], CAST [16], CLICK [17], maximal clique [18-20], and paraclique [21]. These methods use a graph approach, with genes as nodes and edges between genes defined based on a similarity measure. These methods can be divided into two groups: heuristic approaches, and clique-based methods. WGCNA, CAST, and CLICK are all heuristic approaches, computing approximations to various graph-based metrics. The remaining methods (k-clique communities, NNN, maximal clique, and paraclique) depend upon finding cliques, or completely connected subgraphs, but use these resulting cliques in different manners. For example, NNN connects each gene to *n *similar genes and merges overlapping cliques in the resulting graph to form an initial set of networks, while paraclique begins with a clique as a dense core of vertices and gloms on other highly connected genes.

Finally, two other approaches are included: self-organizing maps [22], and QT Clust [23]. SOM is a machine learning approach that groups genes using neural networks. QT Clust is a method developed specifically for expression data. It builds a cluster for each gene in the input, outputs the largest, then removes its genes and repeats the process until none are left.

Many issues influence the selection and tuning of clustering algorithms for gene expression data. First, genes can either be allowed to belong to only one cluster or be included in many clusters. Non-disjoint clustering conforms more accurately to the nature of biological systems, but at a cost of creating hundreds to thousands of clusters. Second, because each method has its own set of parameters for controlling the clustering process, one must determine the ideal parameter settings in practice. There are many different metrics for this problem, which have been evaluated extensively [6,24]. Because there is no way of measuring bias of one metric for a particular clustering method and data set, most clustering comparisons evenly sample the reasonable parameter space for each method [7,25].

There are commonalities between parameters used by some of these methods (shown in Table 1), for example the average cluster size *k *used by hierarchical methods is directly related to the number of clusters required by the k-means algorithm. Other methods such as NNN and QtClust employ readily-computable graph metrics such as minimum neighborhood size and maximum cluster diameter. The clique-based methods all utilize a threshold value, the variation of which often affects the above metrics such as cluster size. Higher threshold values generally result in a less connected graph and therefore smaller cluster sizes. WGCNA relies upon a power parameter, to which correlation values are raised. This results in a form of soft thresholding, where the disparity between low and high correlation values is emphasized. CLICK employs a cluster homogeneity parameter, which is a measure of similarity between items in a cluster. This is related to the idea of using a threshold value, since any two vertices in a threshold-filtered graph are guaranteed to possess a minimum pairwise similarity.

Metrics for comparing clusters can be categorized into two types: internal and external [26]. Internal metrics are based on properties of the input data or cluster output, and are useful in determining how or why a clustering method performs as it does. It provides a data-subjective interpretation that is typically only relevant to a single experimental context. Examples of internal metrics include average correlation value, Figure of Merit (FOM) [27], or diameter [23] which are difficult to compare. External metrics, on the other hand, provide an objective measure of the clusters based on data not used in the clustering process, such as biological annotation data. An external metric does not depend on the experimental context that produced it. Such metrics enable a comparison of the relative merits of these algorithms based on performance in a typical biological study, and can be compared regardless of the annotation source.

External metrics have been used in many previous studies of clustering performance. Some comparisons use receiver operating characteristic (ROC) or precision-recall curves [5]. These metrics are simple to calculate, but they provide too many dimensions (two per cluster) for a straightforward comparison of the overall performance of the methods. While it is possible to give an area under curve (AUC) summary of the entire dataset, this is often not useful to an experimentalist. A full ROC plot would cover every single cluster produced, though the majority of these would normally not be considered. Many other studies [7,25,27,28] have used the Rand Index [29], which generates a single value to measure similarity of clustering results to a known categorization scheme such as GO annotation [30]. However, it is subject to many sources of bias, including a high number of expected negatives typically confirmed when comparing clustering results to categorized gene annotations [5,26]. The Jaccard similarity coefficient ignores true negatives in its calculation, resulting in a measure less dominated by the size of the reference data, particularly in the large number of true negatives that are often confirmed. This idea has been raised in early work using the Rand Index and other partition similarity measures [31] and in the context of comparing clustering algorithms [1]. The Jaccard coefficient has not been widely adopted, due in part to the historical sparseness of annotations in reference sets for comparison. With deep ontological annotation now more widely available, however, external metrics such as the Jaccard coefficient provide a much more relevant, objective, and simplified basis for comparison.

A variety of tools are available to calculate functional enrichment of biological clusters [32-34]. Most are not suitable for high-throughput genome-scale analysis due to interface, speed or scalability limitations. (They often cannot easily handle the large number of clusters produced from whole genome clustering.) Several of the tools are meant exclusively for Gene Ontology terms, precluding the use of the large variety of publicly available annotation sources.

In the present study, we perform an evaluation of both combinatorial and conventional clustering analyses using an evenly distributed parameter set and biological validation performed using Jaccard similarity analysis of KEGG and GO functional gene annotations. For this analysis we implemented a new enrichment analysis tool, specifically designed to handle genome-scale data (and larger) and any gene category annotation source provided. Results of clustering algorithms were compared across all parameters and also in a manner that simulates use in practice by selection of the optima generated from each method.

## Methods

### Data

*Saccharomyces cerevisiae *was fully sequenced in 1996 [35] and has been extensively studied and annotated since. It is therefore an ideal source for biological annotation. We compared the performance of the selected clustering techniques using the extensively studied gene expression data set from Gasch et al. [36]. This data was created to observe genomic expression in yeast responding to two DNA-damaging agents: the methylating agent methylmethane sulfonate (MMS) and ionizing radiation. The set includes 6167 genes from seven yeast strains, collected over 52 yeast genome microarrays.

The microarray data for yeast gene expression across the cell cycle was obtained from http://www-genome.stanford.edu/mec1. These data are normalized, background-corrected, log2 values of the Cy5/Cy3 fluorescence ratio measured on each microarray. We performed clustering either directly on this preprocessed data or on the correlation matrix computed from the data. In the latter case, correlations for gene pairs with five or fewer shared measurements were set to zero. The absolute values of Pearson's correlation coefficients were used, except when a particular clustering approach demands otherwise.

### Clustering methods

In order to evaluate a wide spectrum of approaches likely to be used in practice, and to avoid the difficult task of choosing the arbitrary "best" parameter setting, we selected roughly 20 evenly distributed combinations of reasonable parameter settings for each implementation. To facilitate comparison, we reduced the myriad of output formats to simple cluster/gene membership lists, grouped into three sizes (3-10, 11-100, and 101-1000 genes). For example, hierarchical clustering produces a tree of clusters, which we simply "slice" at a particular depth to determine a list of clusters.

To generate results that were used in scoring, CAST, QT Clust and SOM were run with MeV 4.1 [37]. When running the CAST method, we selected threshold values evenly distributed between 0.5 and 1.0. For QT Clust, we selected maximum cluster diameters evenly distributed from 0.05 to 1.00. SOM requires several parameters, making it difficult to select a narrow parameterization range. We therefore restricted testing to *a × a *grids, choosing 11 values so that the numbers of clusters mirrored the desired cluster sizes. We then ran each of these sizes for both rectangular and hexagonal topologies.

CLICK was run as implemented in Expander4 [17], and we provided homogeneity values evenly distributed from 0.05 to 1.00. k-means and hierarchical clustering were run using the R statistical package [38], with 20 different cluster sizes. Default values for k-means were altered so that the method iterated until convergence and so that each run was repeated ten times *(nstart = 10)*. For NNN, we used publicly available software [15] and varied the minimum neighborhood size from 10-30 (the default is 20). A standalone software package was also used for WGCNA [14]. We applied ten different powers (2, 4, 6, 8, 10, 14, 18, 22, 26, and 30) across two different module detection methods (dynamic height branch cutting and dynamic hybrid branch cutting) and set the minimum module size to 3 (default is 10). WGCNA requires a significance measure for each gene, which was set to 1 for all genes.

Our own implementations were employed for maximal clique and paraclique runs. For maximal clique, we used our highly efficient implementation of the well-known algorithm of Bron and Kerbosch [19], executed on graphs at 21 threshold values evenly distributed from 0.80 to 0.90. For paraclique, we applied four threshold values (0.50, 0.60, 0.70, and 0.80) with five glom factors each (1, 3, 5, 7, and 9).

For *k*-clique communities, we employed the publicly available CFinder software [39]. CFinder produced results in a matter of seconds for thresholds as high as 0.90, but failed to halt within 24 hours for thresholds 0.85 and below. To speed computation, we created our own implementation of the k-clique communities algorithm, using our maximal clique codes. We verified the software by confirming CFinder's clusters at threshold 0.90. We ran our implementation at thresholds 0.80, 0.85, and 0.90, selecting eight values for parameter *k *at each threshold, and evenly distributing them between three and the maximum clique size for that threshold.

Three methods (*k*-means clustering, hierarchical clustering and SOM) required specification of the number of clusters desired. We selected 20 values based on average cluster size, as computed from the total number of genes (6167) divided by number of clusters. We chose two size intervals, 10-100 genes and 100-500 genes, and then selected ten evenly distributed cluster sizes per interval.

### Comparison metrics

Given the prevalence of publicly available gene annotation information, we compared the computationally-derived clusters with manually-curated annotations. Yeast annotation sources include Gene Ontology [30], KEGG Pathways [40], PDB [41], Prosite [42], InterPro [43] and PFAM [44]. For clarity and brevity, and to take advantage of their evenly distributed annotation sizes, the results presented here employ only the Gene Ontology and KEGG Pathways as sources.

We used Jaccard similarity as the basis for our analysis. It is easy to calculate, and concisely compares clusters with a single metric. Jaccard similarity is usually computed as the number of true positives divided by the sum of true positives, false positives, and false negatives.

$$JaccardSimilarity\;=\frac{TruePositives}{TruePositives+FalsePositives+FalseNegatives}$$

In the case of cluster comparisons, this equates to the number of genes that are both in the cluster and annotated, divided by the total number of genes that are either in the cluster or annotated.

$$JaccardSimilarity_{cluster}=\frac{Genes_{cluster}\cap Genes_{annotation}}{Genes_{cluster}\cup Genes_{annotation}}$$

Thus Jaccard measures how well the clusters match sets of co-annotated genes, from 0 meaning no match to 1.0 meaning a perfect match.

We implemented a simple parallel algorithm to search all annotation sources for the genes in each cluster. For each annotation source, we found all annotations that match at least 2 genes in a given cluster. We then computed the number of genes in the cluster that match the annotation (true positives), the number of genes with the annotation but not in the given cluster (false negatives), and the number of genes in the cluster that did not match the annotation (false positives). We ignored genes in the cluster not found in the annotation source. Finally, the highest matching Jaccard score and annotation is assigned to the cluster.

We grouped Jaccard computations by method and parameter settings and then separated each grouping into three cluster size bins: 10 or fewer genes ("small"), 11-100 genes ("medium"), and 101-1000 genes ("large"). When running a clustering algorithm to validate a hypothesis, one generally has an idea of the desired cluster size, which we try to account for with these size classifications. A researcher looking for small clusters is often not interested in a method or tuning that produces large clusters, and vice versa. It is important to note that the use of average cluster size to determine cluster number (as is needed in k-means, hierarchical and SOM clustering) does not mean that all clusters will be of average size. Thus we find that these methods still generate clusters with small, medium and large sizes.

Each individual cluster was scored against the entire annotation set, and the highest Jaccard score match was returned for that cluster. This list of scores was then grouped by cluster size and sorted by Jaccard score. The highest 5 Jaccard scores per cluster size class were averaged to get the Average Top 5 (AT5). This process was then repeated for each parameter setting tested, amassing a list of around 20 AT5 scores per size class. From each list of AT5 scores, the largest value was selected and assigned to the Best Average Top 5 (BAT5) for that size class. This process is then applied to the next clustering algorithm's results. When all data has been collected, the BAT5 scores are output to a summary table, averaged, and sorted again (Table 2).

**Table 2:** Algorithms ranked by quartile comparisons

	Average Quartile	Small (3-10 genes)		Medium (11-100 genes)		Large (101-1000 genes)	
		
Clustering Method		Quartile	BAT5 Jaccard	Quartile	BAT5 Jaccard	Quartile	BAT5 Jaccard
K-Clique Communities	1.00	1	0.7531	1	0.4465	1	0.4915
Maximal Clique	1.00	1	0.8433	1	0.4081		0.0000
Paraclique	1.00	1	0.7576	1	0.4285	1	0.4169
Ward (H)	1.33	2	0.5782	1	0.4011	1	0.5723
CAST	1.67	1	0.7455	3	0.3146	1	0.4994
QT Clust	2.00	2	0.5473	2	0.3670	2	0.3944
Complete (H)	2.33	3	0.3933	2	0.3677	2	0.3419
NNN	2.67	2	0.5521	2	0.3705	4	0.2406
K-Means	3.00	4	0.2573	3	0.3015	2	0.3463
SOM	3.00	4	0.3260	2	0.3286	3	0.3282
WGCNA	3.00	3	0.4391	3	0.3106	3	0.2949
Average (H)	3.33	3	0.4087	4	0.2792	3	0.3037
McQuitty (H)	3.33	3	0.4594	3	0.3065	4	0.2868
CLICK	4.00	4	0.0339	4	0.1453	4	0.2817

**Figure 1:**
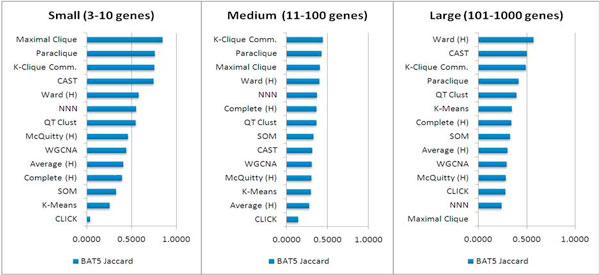
**Algorithms ranked by best average top 5 clusters**. BAT5 Jaccard values are shown for each clustering method and cluster size classification. (H) = Hierarchical clustering agglomeration method.

Results from Figure 1 are displayed by quartile (1 = top 25% - 4 = bottom 25%), with missing values for maximal clique discarded. (H) denotes Hierarchical Clustering agglomeration method.

Clusterings produced by each method were compared pairwise using the variation of information metric [45]. A custom Python script was created to compute this metric, which measures the difference in information between two clusterings. Minimum and maximum variation of information values were extracted for each method pair (additional file 1). This comparison is not applicable to methods with non-disjoint clusters such as maximal clique, so this method was excluded from the analysis.

### Timings

Execution of each method at the parameters producing the optimal BAT5 score for each size class was timed. All timings were generated on a Intel Xeon X5550 2.66 GHz quad core workstation with 12 GB main memory, running 64-bit Ubuntu Linux version 10.04. Methods implemented in the MeV GUI reported run times, while R-based methods were timed using the system.time() function within R. Stand-alone programs paraclique, maximal clique, and k-clique communities were timed using the Unix time function, with the total elapsed time reported by the system being reported. When possible, data load time was excluded. However, this was not possible with several of the methods tested. Similarly, output was suppressed when possible to avoid including the time to write results to disk. The CFinder program allows the specification of various parameters to possibly speed computation of the k-clique communities method such as employing an approximate algorithm and specifying a maximum time to spend for each node, however these options were not used.

## Results

For each clustering method and parameter, clusters of different sizes were obtained. Because there is an exponential distribution of annotation category sizes, matches among small categories are more readily detectable. We binned these results into three size categories (3-10, 11-100, and 101-1000 genes), and ranked the clusters based on Jaccard similarity scores. In practice, users generally are only interested in the few highest-scoring clusters. In many biological studies, only the top 5 to 10 clusters are scrutinized. Data torturing is unfortunately not uncommon in microarray studies due to the wealth of tools available, and in practice, some individuals may perform clustering until a satisfactory result is found. To simulate this practice, we therefore focused on the top five cluster scores for each size grouping (i.e., those with highest Jaccard similarity to annotations), whether derived from match to GO or KEGG annotations, and computed their average score (Average Top 5 or AT5). We chose AT5 as a comparison score because most of the methods produce at least five clusters of each size bin, but for some of the methods, cluster scores drop off quickly after these top five results, making a larger average meaningless. It is also significant that in practice users often adjust parameter settings to improve clusters. Accordingly, for each choice of method and cluster size category, we chose the highest AT5 values across all parameter settings (Best Average Top 5 or BAT5) for that method-size combination. These values are reported in Figure 1. It should be noted that for AT5 and BAT5, maximal clique, like any method that allows non-disjoint cluster membership, creates bias in this score by including results from similar clusters. BAT5 values show that clique-based methods (maximal clique, paraclique, and k-clique communities) perform well, when clusters are compared with available annotation, in all size classes. The CAST method produced high-scoring clusters for small and large groups, and the Ward method of hierarchical clustering was in the top 5 in each case. Most variation was observed at the lower end of the BAT5 scores, though CLICK, along with some hierarchical variants (Average linkage and McQuitty, were consistently low-scoring. Some methods show improved performance in one or more size class. For example, K-Means performed poorly for small and medium clustering, while its BAT5 value was in the upper half of all methods for large clusters.

Another metric of clustering performance is whether a given method is able to find clusters that are readily identified by other methods. This is a direct comparison of the consistency of clustering algorithms. We identified any annotation category that received a Jaccard score greater than 0.25 in any of the hundreds of clustering runs we performed over all parameter settings. This produced a list of 112 annotation categories, 97 from Gene Ontology and 15 from KEGG. We then found the best category match score that each clustering method received on each of these selected annotations and averaged them. Graph based methods scored highest on this internal consistency metric. The best scoring among these were CAST, maximal clique and paraclique. Likewise, the same methods observed to produce low BAT5 values (CLICK, average linkage, and McQuitty) were also unable to identify many genes from these high-scoring annotation categories. It is interesting to note that the K-clique communities method, in contrast to the high BAT5 values produced for all size classes, performed poorly on this metric. For each clustering algorithm, we averaged the BAT5 scores from the three size bins, and ranked them by their average. High-averaging methods not only found good results, but they found them in all three size classifications, indicating robustness to variation in cluster size. These values provide a straightforward way to compare clustering methods irrespective of cluster size.

Figure 3 shows a relative comparison of the number of clusters produced by each clustering method at the optimal parameters for each size class on a log scale. Some methods produced a single cluster at one or more of the size classes. For instance, CLICK generated one cluster in the small and medium size classes while k-Clique Communities produced one cluster in the medium and large size classes. While the maximal clique method did not produce any clusters in the large size class, it produced many more relative to the other methods in the other two size classes. We also examined the average size of the clusters produced, which is displayed in Figure 4. With some variation, all methods produced clusters with a comparable average size. This is possible for the maximal clique method in spite of the much larger number of clusters due to the overlapping nature of the resulting cliques.

To determine the effect of parameter selection on each method, as well as the level of agreement of the various methods over all parameter settings, a comparison of clustering methods using the variation of information metric is presented in additional file 1. Minimum and maximum values are given for variation of information values over all parameters for each pair of methods. Examining the main diagonal, we can see which methods are least affected by changes in input parameters. CLICK, K-Clique Communities, NNN, and QT Clust, for example, all exhibit a small range. Looking at off-diagonal elements can show which methods are very similar or different to one another at some parameter setting. We can observe that many of the hierarchical approaches (Average, Complete, and McQuitty) produce the same set of clustering, indicated by a 0.00 variation of information value, for some parameter selections. This is expected, because for larger k values, very tight clusters will not be merged regardless of the agglomeration strategy used.

Consideration of execution time is also an important aspect when selecting a method for clustering large biological data sets. Table 3 shows the execution time for each of the methods tested at the parameters found to produce the optimal BAT5 Jaccard scores. Most results ranged from just under one minute to more than a minute. Several algorithms such as k-means, QT clust, and k-clique communities took considerably longer. Analysis of algorithm runtimes shows the hierarchical methods to be the fastest. Though graph-based methods are generally expected to take longer to execute, most of these methods finished in roughly 25-80 seconds. Exceptions were paraclique and k-clique communities, with paraclique executing in under 10 seconds for all size classes and k-clique communities unfinished after 2 days.

**Table 3:** Runtimes for each clustering method

	Small (3-10 genes)		Medium (11-100 genes)		Large (101-1000 genes)		
**Clustering Method**	**Param**	**Time (s)**	**Param**	**Time (s)**	**Param**	**Time (s)**	**Implementation**

K-Clique Communities	0.80/03		0.80/57		0.80/48		Standalone,***
Maximal Clique	0.80	26.510	0.80	26.510	N/A	N/A	Standalone,***
Paraclique	0.80/01	5.120	0.80/09	0.780	0.60/09	9.050	Standalone,***
Ward (H)	N/A	2.863	N/A	2.863	N/A	2.863	R 2.13.0
CAST	0.875	37.324	0.85	34.242	0.90	34.121	MeV 4.5.1
QT Clust	030	6 904.518	035	6 759.073	050	5 559.467	MeV 4.5.1
Complete (H)	N/A	2.721	N/A	2.721	N/A	2.721	R 2.13.0
NNN	11	25.550	24	30.610	27	34.370	Standalone,***
K-Means	617	6 711.143	308	4 060.351	21	1 068.069	R 2.13.0
SOM	25/r	6.159	25/h	6.121	18/r	2.956	MeV 4.5.1
WGCNA	2/10	79.430	1/06	80.962	2/06	80.962	R 2.13.0
Average (H)	N/A	2.452	N/A	2.452	N/A	2.452	R 2.13.0
McQuitty (H)	N/A	2.445	N/A	2.445	N/A	2.445	R 2.13.0
CLICK	015	38.270	060	45.310	065	52.570	Standalone,***

Parameters used to produce the best Jaccard score, and the associated runtime for the given method and parameters are displayed. Specific parameter descriptions are listed in Table 1. MeV times were reported by GUI results. Hierarchical methods use the "flashClust" package for R, which is a C++ implementation of the standard "hclust" package. Hierarchical timings do not include the time for tree cutting (which is negligible). flashClust and WGCNA packages were downloaded from the CRAN repository June 22, 2011. Versions reported refer to the version used for runtime calculation; in some cases, previous versions were used to generate clusters for scoring. r = rectangular, h = hexagonal. *A GUI-based graphical tool which is no longer maintained was used to generate clusters for Jaccard scoring while the latest R implementation was using in timing, **Total elapsed time reported by the system.

## Discussion

In our comparison of clustering results by size, we found that maximal clique and paraclique perform best for small clusters; k-clique communities and paraclique perform best for medium clusters; Ward and CAST are the top performing methods for large clusters (Figure 1). Combined analysis of the clustering results across result sizes based upon the quartile of the results reveals that the performance is best for k-clique communities, maximal clique, and paraclique, shown in the first three rows of Table 2.

**Figure 2:**
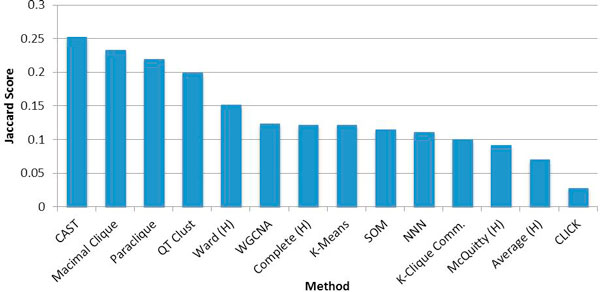
**Algorithms ranked by prominent annotations**. 112 annotations received a Jaccard score above 0.25. Each clustering method was ranked by the average of its highest Jaccard score for each of these annotations. (H) = Hierarchical agglomeration method.

For the analysis of consensus of clustering methods for cluster matches to annotation, we found that CAST, maximal clique and paraclique are best at identifying clustering results found by any other method (Figure 2).

This analysis of the performance of diverse clustering algorithms reveals a performance distinction between graph-based and conventional parametric methods. In our study, the best ranking methods are almost uniformly graph-based, building upon the rigorous cluster definition provided by cliques. Traditional methods suffer from relatively poor performance due to their less rigorous cluster definition or their heuristic nature, which often rely on growth of clusters around individual genes in a neighborhood joining or centroid proximity. These methods do not necessarily result in high inter-correlation among all cluster members, whereas clique and other combinatorial algorithms typically require this by definition.

Although users perform clustering to obtain a decomposition of major co-variation in expression data, conventional clustering algorithms frequently focus on details rather than the bigger picture, by starting from a single gene instead of the full genome. Thus, these methods, such as hierarchical and k-means, lack a global consideration of the data set. Clusters are built incrementally at each step, beginning with a single gene's neighborhood, not a highly correlated geneset. Clusters will therefore tend to converge to a local optimum. This is why repeated randomizations, as is frequently done with k-means, can typically improve results simply by selecting genes with larger neighborhoods. Neural network approaches like SOM suffer from a similar problem, as training takes place with incomplete views of the full data. Even QT Clust suffers from these issues, but to some extent overcomes them through additional computation. Clusters are built incrementally for each gene, but only the highest scoring cluster is partitioned from the rest of the data, at which time its individual genes are partitioned out. The process continues iteratively until no genes remain. Thus, QT clust avoids bias introduced by arbitrarily selecting genes, but still has the same problem of incremental local growth of clusters.

Three of the combinatorial approaches, CAST, CLICK and WGCNA, represent data as graphs, but compute only heuristic solutions to the underlying graph-theoretic metrics. CAST approximates a solution to cluster editing. CLICK approximates a minimum weight cut. WGCNA represents data as a weighted graph, but applies hierarchical clustering to compute its final set of clusters.

NNN computes exact solutions to somewhat arbitrary problem parameters. The poor performance that we observe for it on this data may be because its objectives differ from those of other clustering algorithms. NNN connects genes based on relative correlation among gene pairs. Two genes G and G' are considered related if their correlation is high compared to all other correlations involving G and G', as opposed to all correlations for all genes. Thus, NNN may find clusters that have less pronounced relationships among all cluster members. These clusters may not be present in the high-level GO annotations, but may have biological relevance through more distant and indirect functional relationships.

Variation of information results (additional file 1) indicate that while some methods have a relatively high agreement between individual clustering runs at various parameter settings, there does not seem to be many commonalities between these methods. While some methods such as K-Clique Communities routinely appear in the list of top-scoring methods, others such as CLICK were not able to identify clusters with high-scoring matches to annotation categories. This tells us that consistency over parameter settings is not a good indicator of performance, since many high scoring methods exhibited a wide range of variation over parameter settings. These results further motivate our approach of selecting many common and evenly-spaced parameter settings for our tests.

Our work suggests that graph-based algorithms provide superior reliability and a highly promising approach to transcriptomic data clustering. Most of these methods attempt to find and exploit cliques), with the exception of CLICK which uses minimum cut. It has been suggested that clique-based approaches possess the best potential for identifying sets of interacting genes, due to the highly inter-correlated nature of the clusters produced [46]. The results reported here appear to corroborate that, given that four of the six best performing clustering methods in Table 2 are based upon finding cliques. It should be noted that we applied the algorithms on a single data set for which both deep experimental data and strong biological ground truth are available, and that results may differ when a different data set is used. It is challenging, however, to conceive of a correlation matrix that would be fundamentally biased toward one type of algorithm, especially given that we provided the selection of parameters over a large range of values.

Graph-based problems relevant to clustering are often thought to be difficult to solve (that is, they are *NP*-hard) because globally optimal solutions are required. This can explain both the effectiveness of exact solutions and also why so few algorithms rely on exact solutions. Our results suggest that exact solutions are truly valuable in practice, and that continued research into computing exact solutions to *NP*-hard problems is probably worthwhile.

**Figure 3:**
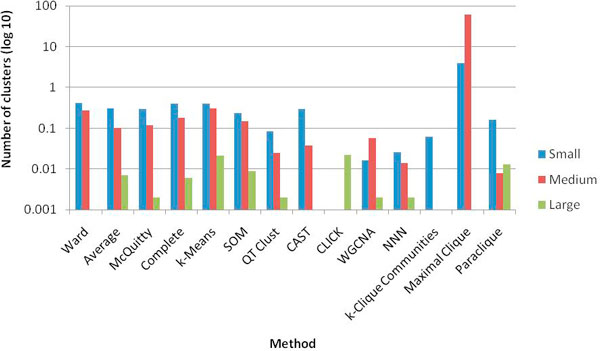
**Number of clusters produced by each method**. The number of clusters produced by each method at the optimal parameter settings for each size class is displayed on a log_10 _scale. Note that some methods produced a single cluster for one or more of the size classes, which appears as absent on the graph. Maximal clique generated no clusters in the large size class, also showing as 0 on the graph.

Though combinatorial approaches to clustering may perform better, implementation challenges have limited widespread adoption to date. Maximal clique's stand-alone utility is rather limited. Even with the best current implementations, it can take a staggering amount of time to run to completion. It tends to overwhelm the user by returning an exhaustive collection of vast numbers of overlapping clusters, even for a small genome such as yeast, which is illustrated in Figure 3. Maximal clique also failed to produce clusters for scoring in the large size class due to its very stringent cluster membership requirement. Paraclique and k-clique communities are appealing alternatives due to the more manageable nature of their results. They employ a form of soft thresholding, which helps to ameliorate the effects of noise and generate nicely-enriched clusters without excessive overlap. From a sea of tangled correlations, they produce dense subgraphs that represent sets of genes with highly significant, but not necessarily perfect, pair-wise correlations. Paraclique relies on maximum clique, and thus operates in a top-down fashion. It generates impressive results through the use of its rigorous cluster definition followed by more lenient expansion, leading to very high average intra-cluster correlations. By avoiding the enumeration problems of maximal clique, it is also highly scalable. Moreover, through its complementary duality with vertex cover, it is amenable to advances in fixed-parameter tractability [47]. Paraclique's main drawback is its use of multiple parameters, making algorithm tuning more challenging. In contrast, k-clique communities relies on maximal clique and so operates in a bottom-up manner. It also generates impressive results, but its dependence on maximal clique severely restricts its scalability. Even for a small genome such as that of *S. cerevisiae*, and even for graphs in which there are no large maximal cliques, we could not run k-clique communities to completion without resorting to our own maximal clique implementation. A faster version of community's CFinder exists (I. Farkas, private comm.), but it achieves speed only by setting timeout values for maximal clique computations, thereby creating an approximation method rather than an optimization method. Thus, given the exponential growth rate of maximal cliques, exact algorithms that rely on such cliques are hobbled by memory limitations on larger genomes and denser correlation graphs. We are rather optimistic, however, that approaches exploiting high performance architectures [20] may have the potential to change this picture. The fourth clique-based approach, this time via cluster editing, is CAST. Although its execution is relatively fast, its heuristic nature ensures only mediocre results and difficult tuning. CAST is simply not an optimization technique. It seems able to detect pieces of important clusters (as evidenced by its prominence in Figure 2), but it is often not comprehensive. Given the extreme difficulty of finding exact solutions to cluster editing [48], we think an optimization analog of CAST is unlikely to be feasible in the foreseeable future.

**Figure 4:**
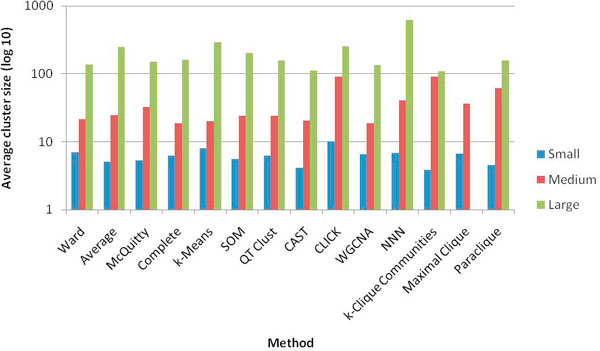
**Average cluster size produced by each method**. The number of clusters produced by each method at the optimal parameter settings for each size class is shown on a log_10 _scale.

Examination of algorithm runtimes put hierarchical methods at the top. However, the performance gain from using graph-based methods appears to come at a runtime cost on the order of seconds. This is particularly true for methods such as paraclique, CAST, NNN, and even maximal clique. With paraclique and maximal clique Jaccard scores falling in the first quartile for all size classes, and CAST in the first quartile for 2 of the 3 size classes, this should be considered further evidence for the utility and applicability of graph-based methods to the clustering of microarray data.

## Conclusions

Using Jaccard similarity to compare clustering results to gene annotation categories, we performed a comparative analysis of conventional and more recent graph-based methods for gene co-expression analyses using a well-studied biological data set. Jaccard similarity provides a simple and objective metric for comparison that is able to distinguish between entire classes of clustering methods without the biases associated with the Rand Index. Our analysis revealed that the best performing algorithms were graph based. Methods such as paraclique provide an effective means for combining mathematical precision, biological fidelity and runtime efficiency. Further development of these sorts of algorithms and of user-friendly interfaces is warranted to facilitate wide-spread application of these techniques among experimentalists.

## Competing interests

The authors declare that they have no competing interests.

## Authors' contributions

JJJ, JDE and YZ produced and collected clusters using the many clustering algorithms. JJJ wrote the software to calculate Jaccard scores and provided the integrative analysis. ADP assisted with analysis, timings and manuscript preparation. MB, AMS, and BHV provided valuable direction for refinement of the results. EJC and MAL conceived of the study and participated in its design, coordination and manuscript preparation. All authors read and approved the final manuscript.

## Supplementary Material

Additional file 1 **Clusterings compared using the variation of information metric**. Results of a pairwise comparison using the variation of information metric is shown. Each entry consists of a minimum and maximum variation of information value of clusters for each pair of methods and selection of parameters. High values indicate very different cluster structures while low values indicate similarity. Values on the main diagonal indicate within-method consistency, with a small range indicating that parameters have little effect on clustering results. Click here for file
