# Supplementary material for: A systematic comparison of genome-scale clustering algorithms
Source: BMC Bioinformatics. 2012 Jun 25;13(Suppl 10):S7. doi: 10.1186/1471-2105-13-S10-S7 (PMC3382433; doi:10.1186/1471-2105-13-S10-S7)
Supplement: Additional file 1 — Clusterings compared using the variation of information metric. Results of a pairwise comparison using the variation of information metric is shown. Each entry consists of a minimum and maximum variation of information value of clusters for each pair of methods and selection of parameters. High values indicate very different cluster structures while low values indicate similarity. Values on the main diagonal indicate within-method consistency, with a small range indicating that parameters have little effect on clustering results. [file 1471-2105-13-S10-S7-S1.pdf]

Additional file 1 - Clusterings compared using the variation of information metric

Results of a pairwise comparison using the variation of information metric is shown. Each entry consists of a minimum and maximum variation of information value of clusters for each pair of methods and selection of parameters. High values indicate very different cluster structures while low values indicate similarity. Values on the main diagonal indicate within-method consistency, with a small range indicating that parameters have little effect on clustering results.

	CAST	CLICK	Average	Complete	McQuitty	Ward	WGCNA	K-Clique Communities
CAST	**(0.00, 25.15)**	(1.46, 17.36)	(0.00, 20.05)	(0.00, 21.35)	(0.00, 20.79)	(3.33, 21.65)	(0.31, 19.57)	(0.09, 13.68)
CLICK	(1.45, 17.36)	**(2.94, 9.33)**	(1.46, 12.23)	(1.46, 13.53)	(1.46, 12.97)	(4.79, 13.83)	(1.77, 11.74)	(1.55 5.86)
Average	(0.00, 20.05)	(1.46, 12.23)	**(0.00, 13.77)**	(0.00, 16.23)	(0.00, 15.66)	(3.33, 16.53)	(0.31, 14.44)	(0.09, 8.55)
Complete	(0.00, 21.35)	(1.46, 13.53)	(0.00, 16.23)	**(0.00, 16.52)**	(0.00, 16.96)	(3.33, 17.83)	(0.31, 15.74)	(0.09 9.85)
McQuitty	(0.00, 20.79)	(1.46, 12.97)	(0.00, 15.66)	(0.00, 16.96)	**(0.00, 15.30)**	(3.33, 17.27)	(0.31, 15.18)	(0.09, 9.29)
Ward	(3.33, 21.65)	(4.79, 13.83)	(3.33, 16.53)	(3.33, 17.83)	(3.33, 17.27)	**(6.75, 17.16)**	(3.64, 16.04)	(3.41, 10.16)
WGCNA	(0.31, 19.57)	(1.77, 11.74)	(0.31, 14.44)	(0.31, 15.74)	(0.31, 15.18)	(3.64, 16.04)	**(0.71, 13.93)**	(0.40, 8.07)
K Clique Communities	(0.09, 13.68)	(1.55, 5.86)	(0.09, 8.55)	(0.09, 9.85)	(0.09, 9.29)	(3.41, 10.16)	(0.40, 8.07)	**(0.19, 2.15)**
K-means	(3.45, 21.78)	(4.92, 13.96)	(3.45, 16.65)	(3.45, 17.96)	(3.45, 17.39)	(6.78, 18.26)	(3.76, 16.17)	(3.54, 10.28)
NNN	(1.48, 17.69)	(2.94, 9.87)	(1.48, 12.57)	(1.48, 13.87)	(1.48, 13.30)	(4.81, 14.17)	(1.79, 12.08)	(1.57 6.19)
Paraclique	(2.90, 21.35)	(4.36, 13.53)	(2.09, 16.22)	(2.90, 17.52)	(2.90, 16.96)	(6.22, 17.83)	(3.21, 15.74)	(2.98 9.85)
QT Clust	(4.35, 20.55)	(5.81, 12.73)	(4.35, 15.43)	(4.35, 16.73)	(4.35, 16.16)	(7.67, 17.03)	(4.66, 14.94)	(4.43 9.05)
SOM	(3.14, 20.35)	(4.61, 12.53)	(3.14, 15.22)	(3.14, 16.52)	(3.14, 15.96)	(6.47, 16.83)	(3.45, 14.74)	(3.23 8.85)

(continued)

	K-Means	NNN	Paraclique	QT Clust	SOM
CAST	(3.45, 21.78)	(1.48, 17.69)	(2.90, 21.35)	(4.35, 20.55)	(3.14, 20.35)
CLICK	(4.92, 13.96)	(2.94, 9.87)	(4.36, 13.53)	(5.81, 12.73)	(4.61, 12.53)
Average	(3.45, 16.65)	(1.48, 12.57)	(2.90, 16.22)	(4.35, 15.43)	(3.14, 15.22)
Complete	(3.45, 17.96)	(1.48, 13.87)	(2.90, 17.52)	(4.35, 16.73)	(3.15, 16.52)
McQuitty	(3.45, 17.39)	(1.48, 13.30)	(2.90, 16.93)	(4.35, 16.16)	(3.14, 15.96)
Ward	(6.78, 18.26)	(4.81, 14.17)	(6.22, 17.83)	(7.67, 17.03)	(6.47, 16.83)
WGCNA	(3.76, 16.17)	(1.79, 12.08)	(3.21, 15.74)	(4.66, 14.94)	(3.45, 14.74)
K Clique Communities	(3.54, 10.28)	(1.57, 6.19)	(2.98, 9.85)	(4.43, 9.05)	(3.23, 8.85)
K-means	**(7.03, 17.39)**	(4.93, 14.30)	(6.35, 17.95)	(7.80, 17.16)	(6.59, 16.95)
NNN	(4.93, 14.30)	(**2.97, 10.17)**	(4.38, 13.86)	(5.83, 13.07)	(4.62, 12.86)
Paraclique	(6.35, 17.95)	(4.38, 13.86)	**(6.07, 17.28)**	(7.25, 16.72)	(6.04, 16.52)
QT Clust	(7.80, 17.16)	(5.83, 13.07)	(7.25, 16.72)	**(9.20, 15.90)**	(7.49, 15.72)
SOM	(6.59, 16.95)	(4.62, 12.86)	(6.04, 16.52)	(7.49, 15.72)	**(6.68, 14.81)**
